# Persistent hypermetabolism and longitudinal energy expenditure in critically ill patients with COVID-19

**DOI:** 10.1186/s13054-020-03286-7

**Published:** 2020-09-28

**Authors:** John Whittle, Jeroen Molinger, David MacLeod, Krista Haines, Paul E. Wischmeyer, for the LEEP-COVID Study Group, John Whittle, Jeroen Molinger, David MacLeod, Krista Haines, Paul E. Wischmeyer, Anthony Sung, Marat Fudim, Lindsie Boerger, Kathryn Lessig, Jessica Lumbard, Leslie C. Murray, Sue Steves, Jhana Parikh, Jacob Ribet, Melanie Hollidge

**Affiliations:** 1 Division of Critical Care, Human Pharmacology and Physiology Laboratory (HPPL), Department of Anesthesiology, Duke University School of Medicine, DUMC, Box 3094 Mail # 41, 2301 Erwin Road, 5692 HAFS, Durham, NC 27710 USA; 2 Division of Trauma Critical Care, and Acute Care Surgery, Department of Surgery, Duke University School of Medicine, Durham, NC USA

**Keywords:** Metabolism, Nutrition, Indirect Calorimetry, SARS-CoV-2, ICU, Metabolic cart, VO2, VCO2

COVID-19 infection results in respiratory failure requiring ICU care in a small, yet significant, number of patients [1]. The longitudinal metabolic phenotype and energy expenditure of this novel pandemic disease has yet to be described. As a marked and often prolonged, systemic inflammatory response (SIRS) has been suggested to be a hallmark of severe COVID-19 infection [1], we hypothesized a prolonged hypermetabolic state would evolve over ICU stay that would persist beyond the 7–10 day hypermetabolic phase described previously in other ICU conditions [2].

Further, understanding the energy expenditure of COVID-19 ICU patients is essential to help determine safe, optimal nutrition needs for the ICU provider [3], as both over-/underfeeding is associated with increased ICU mortality [3, 4]. Prediction of resting energy expenditure (pREE) using standardized formulas or bodyweight calculations often correlates poorly with measured REE (mREE) [3]. Thus, our aim was to assess longitudinal mREE via indirect calorimetry (IC) in intubated COVID-19 patients.

Here, we report the first results from the LEEP-COVID study (clinicaltrials.gov NCT04350073) from March to May, 2020. Following IRB approval, IC was conducted every 72 h (Q-NRG, COSMED/BAXTER, USA) [5]. Prior to testing, patients were confirmed to be in stable condition with only steady-state measures for ≥ 20 min considered valid. mREE was compared to pREE, which was calculated at same timepoints via commonly utilized Harris-Benedict equation (HBE). For calculations, actual body weight (ABW) was used for non-obese (BMI < 30) and both actual and adjusted body weight (AdjBW) was utilized for obese subjects (BMI > 30) [3].

Data from 22 COVID-19 ICU patients are summarized in Table 1 and Fig. 1. During the 1st ICU week, mREE was observed to fall between 15 and 20 kcal/kg (for ABW in BMI < 30 and AdjBW in obese subjects [3].). Increasing hypermetabolism and wider variability in mREE were observed post-1st ICU week. Unlike data from smaller studies in other ICU populations [1], observed hypermetabolism persisted, and in fact increased during 3rd ICU week (mean mREE = 150% pREE in 3rd ICU week). Certain individuals exhibited metabolic rates greater than two-times predicted via HBE, which significantly underpredicted REE post-1st ICU week. Changes in mREE may not be significantly related to severity of organ failure and only minorly affected by paralysis/prone positioning, as these were not significantly different over the study period (Table 1).

**Table 1:** Baseline characteristics, clinical care and outcomes, and indirect calorimetry measured resting energy expenditure in COVID-19 ICU patients

**(a) Baseline characteristics (*n*** **= 22)**				
Age (mean, range)	58 (31–88)			
Male sex (*n*, %)	13 (59)			
Race (*n*, %)				
African-American/Black	12 (54)			
Caucasian/White	7 (32)			
Hispanic	3 (14)			
BMI (mean, range)	30.7 (17.4–48.1)			
BMI > 30 (%)	55			
Ventilator days (21-day study period only) (mean, sd)	14.4 (4.7)			
Mortality (21-day study period only) (*n*, %)	3 (14)			
Mortality (hospital mortality) (*n*, %)	5 (22)			
**(b) Energy expenditure/data**	D0–7	D7–14	D14–21	*p* value
Measured REE in absolute kCal/day (all patients) (median, IQR)	1568 (1175–2215)	1830 (1465–2467)	2789 (1776–3262)	< 0.05
Measured REE kCal/kg actual BW (non-obese, BMI < 30) (median, IQR)	19.2 (16.9–20.7)	26 (24.5–35.5)	29 (23–34.5)	< 0.05
Measured REE kCal/kg actual BW (obese, BMI > 30) (median, IQR)	17.5 (12–19.25)	21 (20–23.5)	31.5 (24.8–36)	< 0.05
Measured REE kCal/kg adjusted BW (obese, BMI > 30) (median, IQR)	20 (17–22.5)	26.3 (24–29)	32.5 (28.8–35.8)	< 0.05
Measured REE kCal/kg actual BW (all patients) (median, IQR)	19 (13.7–28.5)	26 (22–42)	30.4 (27–35.8)	< 0.05
**(c) Clinical data**	D0–7	D7–14	D14–21	*p* value
Use of prone positioning (%) (mean, sd)	12.3 (8.6)	7 (2.4)	12.2 (4.3)	0.17
Use of paralysis with neuromuscular blocker (%) (mean, sd)	14.8 (8)	9.7 (1.7)	12.3 (3.4)	0.2
SOFA score (mean, sd)	9 (3.6)	9 (3.2)	9.5 (3.6)	0.5

a, patient characteristics; b, nutritional data for the first 3 weeks post-intubation; c, clinical care and outcomes data

*BW* body weight; *BMI* body mass index; *REE* resting energy expenditure, predicted REE via Harris-Benedict equation; *AdjBW* adjusted bodyweight, *ABW* actual body weight, *obesity* BMI > 30, *non-obese* BMI < 30I, *IQR* interquartile range, *SOFA* Sequential Organ Failure Assessment, *sd* standard deviation

Notes: All obese subjects had BMI measures between 30 and 50. *p* values are for Kruskal-Wallis test

Subjects were withdrawn from this analysis upon extubation or death

**Fig. 1:**
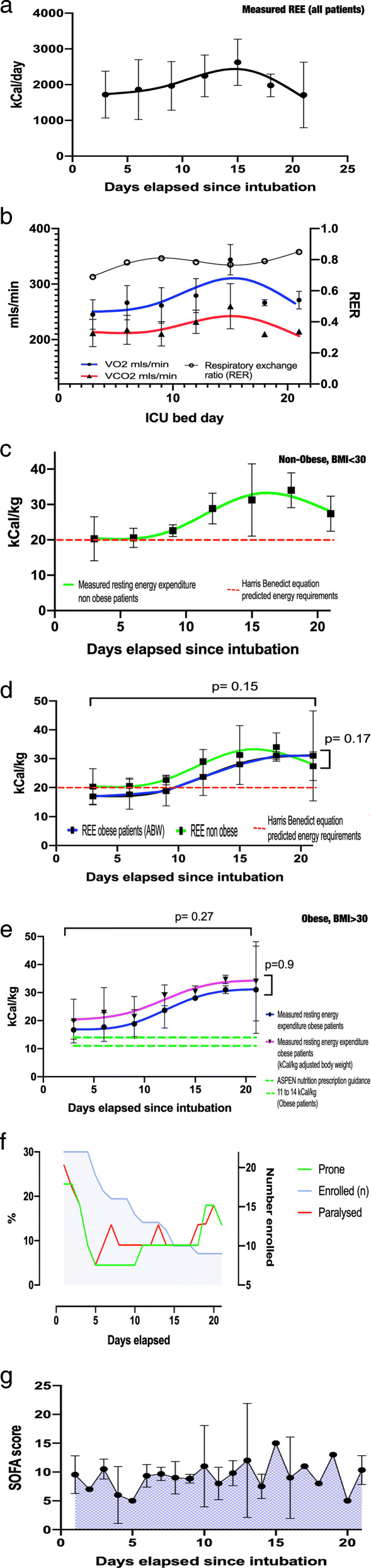
Longitudinal energy expenditure, VO2/VCO2, and clinical care and SOFA score in intubated COVID-19 ICU patients over first 3 ICU weeks post-intubation. **a** Resting absolute energy expenditure over time in intubated patients with SARS-CoV-2 infection. **b** Resting VO_2_, VCO_2_, and RER over time in intubated patients with SARS-CoV-2 infection. **c** Resting energy expenditure over time in intubated non-obese patients with SARS-CoV-2 infection. **d** Resting energy expenditure over time in intubated non-obese and obese patients with SARS-CoV-2 infection. **e** Resting energy expenditure over time in intubated obese patients with SARS-CoV-2 infection. **f** Percentage (%) of intubated patients with SARS-CoV-2 who were in prone position or paralyzed with neuromuscular blockers throughout study. Total number of subjects at any time point still participating in the study is presented in blue. **g** SOFA scores over time in intubated patients with SARS-CoV-2. Notes: (i) Longitudinal data presented as fitted regression curves (locally weighted scatter plot smoothing, with a 10-point smoothing window) with 72 h measured REE values presented as mean (sd), *p* values are for MANOVA comparing both differences over time (longitudinal) and at individual time points. (ii) Metabolic cart measurements were able to be conducted on patients with an FiO2 < 70% per manufacturer specifications and as described in new generation metabolic cart (QNRG) validation study reference [5]. (iii) Abbreviations: VO2, volume of oxygen consumed per minute; VCO2, volume of carbon dioxide consumed per minute; obesity, BMI > 30–50, non-obese, BMI < 30; REE, resting energy expenditure; kCal, kilocalories; RER, respiratory exchange ratio; ASPEN, American Society for Parenteral and Enteral Nutrition

Longitudinal IC data presented here demonstrate a progressive hypermetabolic phenotype beginning 1 week post-intubation in COVID-19 ICU patients, with significantly greater mREE versus predictive equations or ASPEN-recommended 11–14 kcal/kg ABW for obese subjects used currently to determine energy requirements. Our data support use of standard predictive equations or ~ 20 kcal/kg as a reasonable approximation of mREE in 1st ICU week in COVID-19 patients. Current ESPEN/ASPEN ICU guidelines suggest hypocaloric (~ 70% pREE) feeding during acute phase to prevent overfeeding risk as it is believed ICU patients have initial early endogenous nutrient production that we currently are unable to measure [3, 4].

To our knowledge, this is the first description of longitudinal mREE in a COVID-19 ICU population. The COVID-19 metabolic phenotype may be unique from previously described ICU models of metabolic response [2], with a more prolonged hypermetabolic phase that may be independent of severity of organ failure and, as previously published, may only be minorly affected by interventions such as paralysis [6]. Further, it is one of the largest single-ICU diagnosis cohorts with longitudinal IC measures for 21 days. In conclusion, we demonstrate progressive hypermetabolism and considerable variation in REE throughout ICU stay. We hope this data assists ICU clinicians in further understanding the effects of COVID-19 on metabolism and in assessing nutrition care needs. These data suggest personalization of nutrition delivery, including IC use [3, 5], should be considered to provide more accurate assessments of energy expenditure and help guide nutrition delivery in COVID-19 ICU patients.

## Data Availability

All raw data available upon request

## References

[1] Berlin DA, Gulick RM, Martinez FJ. Severe Covid-19 [published online ahead of print, 2020 May 15]. N Engl J Med. 2020; 10.1056/NEJMcp2009575. 10.1056/NEJMcp2009575 32412710

[2] Uehara M, Plank LD, Hill GL (1999). Components of energy expenditure in patients with severe sepsis and major trauma: a basis for clinical care. Crit Care Med.

[3] Singer P, Blaser AR, Berger MM, Alhazzani W, Calder PC, Casaer MP, Hiesmayr M, Mayer K, Montejo JC, Pichard C (2019). ESPEN guideline on clinical nutrition in the intensive care unit. Clin Nutr.

[4] McClave SA, Taylor BE, Martindale RG, Warren MM, Johnson DR, Braunschweig C, McCarthy MS, Davanos E, Rice TW, Cresci GA (2016). Guidelines for the provision and assessment of nutrition support therapy in the adult critically ill patient: Society of Critical Care Medicine (SCCM) and American Society for Parenteral and Enteral Nutrition (A.S.P.E.N.). JPEN J Parenter Enteral Nutr.

[5] Oshima T, Delsoglio M, Dupertuis YM, Singer P, De Waele E, Veraar C, Heidegger CP, Wernermann J, Wischmeyer PE, Berger MM et al: The clinical evaluation of the new indirect calorimeter developed by the ICALIC project. Clin Nutr. 2020:S0261-5614(20)30040-6. Doi: 10.1016/j.clnu.2020.01.017. Online ahead of print. 10.1016/j.clnu.2020.01.017 32046881

[6] Koekkoek WAC, Menger YA, van Zanten FJL, van Dijk D, van Zanten ARH (2020). The effect of cisatracurium infusion on the energy expenditure of critically ill patients: an observational cohort study. Crit Care.

